# Leprosy and gender in Brazil: trends in an endemic area of the Northeast region, 2001–2014

**DOI:** 10.11606/S1518-8787.2018052000335

**Published:** 2018-02-07

**Authors:** Eliana Amorim de Souza, Anderson Fuentes Ferreira, Reagan Nzundu Boigny, Carlos Henrique Alencar, Jorg Heukelbach, Francisco Rogerlândio Martins-Melo, Jaqueline Caracas Barbosa, Alberto Novaes Ramos

**Affiliations:** I Universidade Federal da Bahia. Instituto Multidisciplinar em Saúde, Campus Anísio Teixeira. Núcleo Epidemiologia e Saúde Coletiva. Vitória da Conquista, BA, Brasil; II Universidade Federal do Ceará. Faculdade de Medicina. Programa de Pós-Graduação em Saúde Pública. Fortaleza, CE, Brasil; III Universidade Federal do Ceará. Faculdade de Medicina. Departamento de Saúde Comunitária. Fortaleza, CE, Brasil; IV James Cook University. College of Public Health, Medical and Veterinary Sciences. Division of Tropical Health and Medicine. Townsville, Queensland, Australia; V Instituto Federal de Educação, Ciência e Tecnologia do Ceará. Caucaia, CE, Brasil

**Keywords:** Leprosy, epidemiology, Gender and Health, Disease Notification, Endemic Diseases, Neglected Diseases, Time Series Studies, Hanseníase, epidemiologia, Gênero e Saúde, Notificação de Doenças, Doenças Endêmicas, Doenças Negligenciadas, Estudos de Séries Temporais

## Abstract

**OBJECTIVE:**

To analyze, stratifield by gender, trends of the new case leprosy detection rates in the general population and in children; of grade 2 disability, and of proportion of multibacillary cases, in the state of Bahia, Brazil from 2001 to 2014.

**METHODS:**

A time series study based on leprosy data from the National Information System for Notifiable Diseases. The time trend analysis included Poisson regression models by infection points (Joinpoint) stratified by gender.

**RESULTS:**

There was a total of 40,054 new leprosy cases with a downward trend of the overall detection rate (Average Annual Percent Change [AAPC = -0.4, 95%CI -2.8–1.9] and a non-significant increase in children under 15 years (AAPC = 0.2, 95%CI -3.9–4.5). The proportion of grade 2 disability among new cases increased significantly (AAPC = 4.0, 95%CI 1.3–6.8), as well as the proportion of multibacillary cases (AAPC = 2.2, 95%CI 0.1–4.3). Stratification by gender showed a downward trend of detection rates in females and no significant change in males; in females, there was a more pronounced upward trend of the proportion of multibacillary and grade 2 disability cases.

**CONCLUSIONS:**

Leprosy is still highly endemic in the state of Bahia, with active transmission, late diagnosis, and a probable hidden endemic. There are different gender patterns, indicating the importance of early diagnosis and prompt treatment, specifically in males without neglecting the situation among females.

## RESUMO

**OBJETIVO:**

Analisar, segundo gênero, as tendências temporais dos coeficientes de detecção de casos novos de hanseníase na população geral e em crianças, do coeficiente de grau 2 de incapacidade física e da proporção de casos multibacilares no estado da Bahia, no período de 2001 a 2014.

**MÉTODOS:**

Estudo de série temporal baseado em dados epidemiológicos relativos à hanseníase oriundos do Sistema de Informação de Agravos de Notificação. A análise de tendência temporal incluiu modelos de regressão de Poisson por pontos de inflexão (*Joinpoint*), considerando-se a dimensão de gênero.

**RESULTADOS:**

Dos 40.054 casos notificados no período, verificou-se tendência de redução no coeficiente de detecção geral (*Average Annual Percent Change* [AAPC] = -0,4; IC95% -2,8–1,9) e aumento em menores de 15 anos sem significância estatística (AAPC = 0,2; IC95% -3,9–4,5). Para o coeficiente de detecção com grau 2 de incapacidade física, a tendência foi de aumento significativo (AAPC = 4,0; IC95% 1,3–6,8), assim como nos casos multibacilares (AAPC = 2,2; IC95% 0,1–4,3). A análise estratificada por gênero indicou tendência de redução dos coeficientes de detecção entre as mulheres e de manutenção entre os homens, sem significância estatística. Em relação ao coeficiente de detecção em menores de 15 anos, a tendência de redução é mais acentuada nas mulheres do que entre os homens. As mulheres apresentaram tendência mais acentuada de aumento na proporção de casos multibacilares e do coeficiente de detecção de grau 2 de incapacidade.

**CONCLUSÕES:**

O estado da Bahia mantém alta endemicidade, transmissão ativa, diagnóstico tardio e provável endemia oculta de hanseníase. Há um padrão diferencial segundo gênero, tornando central o diagnóstico e tratamento oportuno com estratégias diferenciadas para os homens, sem desconsiderar a situação entre as mulheres.

## INTRODUCTION

Leprosy is a chronic condition with high potential for causing disability and stigma perpetuating the vicious circle of poverty^1^ ^,^ ^21^. A significant decrease of incidence rates after introduction of the multidrug therapy can be observed, but the disease is still a cause of morbidity, especially in vulnerable populations ^3^ ^,^ ^6^ ^,^ ^23^ ^,^ ^28^ ^,^ ^29^. In addition, the impact on mortality patterns from direct or indirect causes related to the disease is present, especially in the male population^18^ ^,^ ^19^.

Leprosy cases are not distributed homogeneously in Brazil, with areas of higher risk mainly located in the Midwest, North, and Northeast regions^3^ ^,^ ^21^. The ten clusters with the highest risk of occurrence of new cases (NC) are located mainly in the states of Mato Grosso, Pará, Maranhão, Tocantins, Goiás, Rondônia, and Bahia; together, they account for 44% of all cases diagnosed in 2013^21^. In 2014, the state of Bahia ranked thirteenth in the national ranking of new case detection rates with 17.4 new leprosy cases per 100,000 inhabitants, higher than the Brazilian average (15.3 cases per 100,000 inhabitants)^a^.

The Integrated Plan of Strategic Actions for the elimination or significant reduction of the burden caused by some Neglected Tropical Diseases in Brazil, published in 2012, defined priority municipalities and established active case finding among intradomiciliary and school contacts as important intervention measures^b^. In 2016, the specific guidelines for the surveillance, treatment, and elimination of leprosy were updated, re-discussing health promotion and education measures, active case finding for early diagnosis, prompt treatment, prevention, and treatment of physical disabilities and rehabilitation, and contact tracing, as well as guidelines for BCG vaccination (*Bacillus Calmette-Guérin*)^c^. These new guidelines emphasized the importance of adopting epidemiological indicators to monitor the progression of leprosy as a public health problem, as well as operational indicators to evaluate the performance of health services.

In the new guidelines of 2016, three new indicators were established; among them, the gender distribution among the number of new cases detected^c^. Similar to other Neglected Tropical Diseases, leprosy is not only associated with poverty, but it also shows a specific gender distribution in terms of morbidity and mortality^26^ ^,^ ^27^.

Depending on the socio-cultural context, gender distribution varies. Gender inequalities verified from the sociocultural point of view in the Brazilian reality, as well as in Latin America, are strongly accentuated. In fact, differences in morbidity and mortality between men and women can be socially determined by lifestyle, customs, habits, and social behaviors^15^ ^,^ ^26^ ^,^ ^27^.

Based on the assumption that leprosy affects men and women differently in Brazil^a^, epidemiological studies including the gender perspective are important for planning the implementation of control measures in the perspective of integral health and equity. The objective of this study was to analyze the temporal trends of the new case detection rates of leprosy in the general population and in children under 15 years, the detection rate with grade 2 disability (G2D), and the proportion of cases with multibacillary (MB) leprosy among new cases, according to gender, in the state of Bahia, from 2001 to 2014.

## METHODS

### Study Area

**Figure 1:**
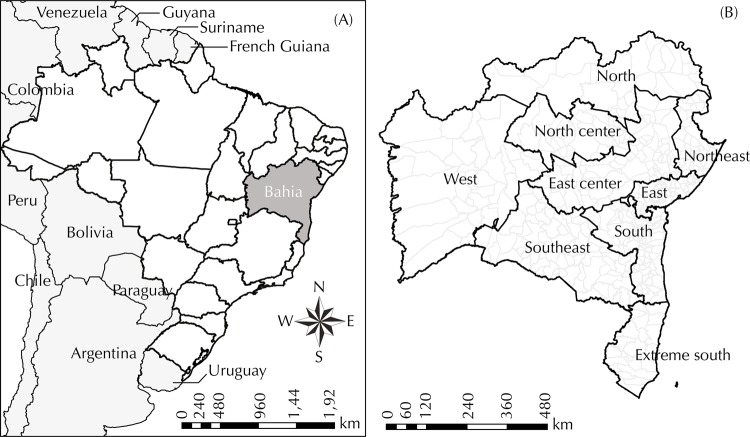
Health regions of the State of Bahia, Northeast region of Brazil.

The state of Bahia is the largest (564,830,859 km^2^) and most populated (15,203,934 inhabitants in 2015) state of Brazil’s Northeast region^d^. Its 417 municipalities are administratively distributed, into nine Regional Health Centers (Figure 1).

The state ranks eighth nationwide in terms of Gross Domestic Product (GDP) and first in the Northeast region. However, with a Human Development Index (HDI) of 0.660 in 2014, it is in the average range of development^d^. According to the FIRJAN Municipal Development Index (FMDI) published in 2015, which integrates the areas of Employment and Income, Education, and Health for 2013, the municipalities of Bahia show a very unfavorable socioeconomic situation as compared to most Brazilian municipalities from other states. While 68.1% of the Brazilian municipalities presented high or moderate development, only 11.8% of the municipalities of Bahia were classified in this group. Of the 100 lowest FMDI of the country, 41 were registered in the municipalities of Bahia, and among the more than 500 least developed municipalities in Brazil, 178 were located in Bahia^e^. In this sense, the state is marked by deep inequalities, with a strong concentration of income, occupying the fourth position in the country in terms of the Gini index, with a value of 0.559^f^.

### Study Design and Data Sources

This is a time series study based on secondary data of new cases of leprosy that resided in the state of Bahia from 2001 to 2014, at the time of diagnosis. The data source was the Information System of Notifiable Diseases – Ministry of Health (SINAN) of the Brazilian Ministry of Health, whose database was obtained from the State of Bahia (State Health Department). Cases with “diagnostic error” as output criteria were excluded. Population data were obtained from the Brazilian Institute of Geography and Statistics (IBGE) based on the demographic census of the state (2010), as well as on the population estimates for intercensal years (2001 to 2009 and 2011 to 2014).

### Statistical Analysis

We included the following variables in the descriptive analysis: age, gender, ethnicity/race, and educational level. To describe the epidemiological patterns of leprosy according to gender (which allows the verification of the capacity of the services to attend the cases of leprosy), we calculated the following indicators: annual new case detection rate in the general population per 100,000 inhabitants (which measures the strength of the morbidity, magnitude, and endemic trend), annual new case detection rate in children under 15 years per 100,000 inhabitants (which measures the strength of the recent endemic transmission and its trend), rate of leprosy cases with G2D at diagnosis per 100,000 inhabitants (which are deformities caused by leprosy in the general population and which allows the comparison with other incapacitating diseases), and finally, the proportion of cases with MB leprosy among new cases (indicating cases at risk of developing complications and the correct reestablishment of multidrug therapy)^c^. We used Tabwin^®^ software, version 3.6 (Department of Informatics of the Brazilian Unified Health System – DATASUS) for data extraction and descriptive analysis. We considered the parameters of the Ministry of Health to evaluate the indicators^c^.

For the time trend analysis, we used the indicators for the state and, then, we performed the Poisson Joinpoint regression using the Joinpoint Regression Program, version 4.4.2 (http://surveillance.cancer.gov/joinpoint/). This statistical technique provides the adjustment of a series of lines, as well as their inflection points, on a logarithmic scale using the annual trends test. To obtain the adjustment based on the best line of each analyzed segment, we used the Monte Carlo permutation method as a test of significance. From the definition of the follow-ups, we estimated and tested the annual percentage change (APC) and the average annual percentage change (AAPC), with their respective 95% confidence intervals (95%CI). If we identified the occurrence of an inflection point with inverted direction, we analyzed the study periods separately. In the same way, the number of inflections used in the analysis represented the result of models defined by the program itself, in order to allow a better representation of the trend, with the lowest number of inflection points. The result showed an increase (positive APC values), decrease (negative APC values), or maintenance (APC value equal to zero) of the trend throughout the whole historical series (2001–2014)^12^.

The study respects the Resolution of the National Health Council number 466, dated October 12, 2012, and has been approved by the Ethical Review Board of the Universidade Federal do Ceará (Opinion 544.962 of February 28, 2014). All data used in this article are of public access and domain.

## RESULTS

In the study period, 40,054 new cases of leprosy were reported, with a higher proportion of males (50.3%), illiterate persons or who studied until the incomplete fourth grade (34.8%), brown (51.9%), and individuals aged between 30 and 44 years (24.7%).

**Figure 2:**
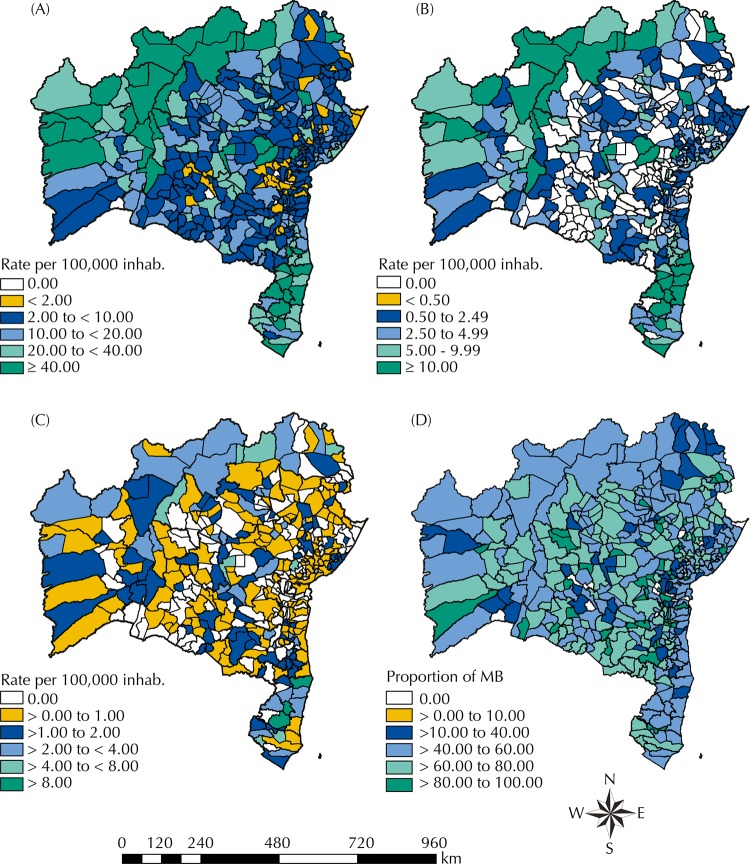
Spatial distribution of the general detection rates of new cases of leprosy per 100,000 inhabitants (A), detection rate in children < 15 years per 100,000 inhabitants (B), detection rate with grade 2 disability at diagnosis per 100,000 inhabitants (C), and proportion of multibacillary cases among new cases (D). Bahia, Brazil, 2001–2014.

Only five municipalities did not report any cases throughout the fourteen-year period. Of the municipalities with cases of leprosy, 48.7% (n = 201) were classified as having an average endemicity and 8% (n = 33) as hyperendemic (Figure 2, A). Cases with children < 15 years occurred in 56.1% (n = 234) of the municipalities, of which, 26.0% (n = 61) were classified as hyperendemic (Figure 2, B). Cases of G2D occurred in 65.0% (n = 271) of the municipalities (Figure 2, C). Approximately 60% of the municipalities in Bahia presented at least one confirmed MB case (Figure 2, D).

MB: multibacillary cases

**Figure 3:**
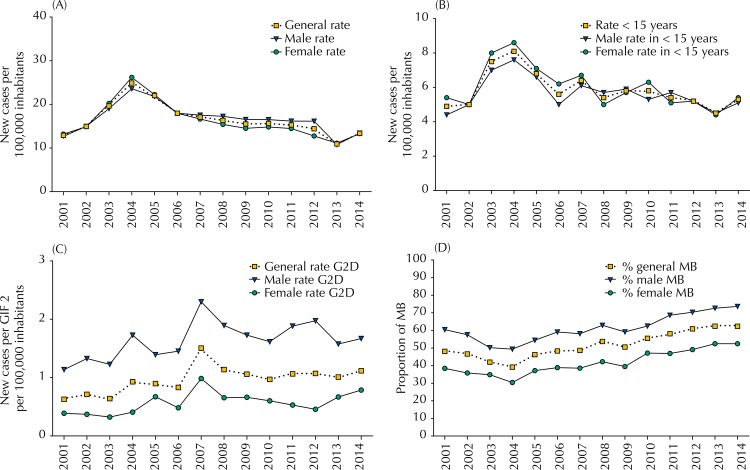
Historical series of epidemiological indicators of leprosy by gender: general detection rates of new cases of leprosy per 100,000 inhabitants (A), detection rate in children under 15 years per 100,000 inhabitants (B), detection rate with grade 2 disability at diagnosis per 100,000 inhabitants (C), and proportion of multibacillary cases among new cases (D). Bahia, Brazil, 2001–2014.

The overall new case detection rate in 2014 was 17.4 cases/100,000 inhabitants. Throughout the study period, the mean of this indicator was 20.4 cases/100,000 inhabitants and, after 2004 (28.7 cases/100,000 inhabitants) (Figure 3, A), there was a statistically significant decrease over time (APC = -4.9, 95%CI -6.5– -3.4). Throughout the historical series, AAPC was -0.4 (95%CI -2.8–1.9), indicating maintenance of the trend (Table).

G2D: grade 2 disability, MB: multibacillary cases

**Table:** Trend of the epidemiological indicators of leprosy, according to Joinpoint model stratified by gender. Bahia, Brazil, 2001–2014.

Indicator	Gender	Trend 1			Trend 2			Entire Period		Trend line
		
		Period	APC	95%CI	Period	APC	95%CI	AAPC	95%CI	
General detection rate ^b^	M	2001–2004	16.5 ^a^	2.8–32.0	2004–2014	-4.5 ^a^	-6.3– -2.6	-0.0	-2.8–2.8	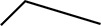
	F	2001–2004	15.9 ^a^	3.8–29.4	2004–2014	-5.5 ^a^	-7.1– -3.9	-0.9	-3.4–1.6	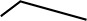
	General	2001–2004	16.1 ^a^	4.6–28.9	2004–2014	-4.9 ^a^	-6.5– -3.4	-0.4	-2.8–1.9	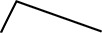
Detection rate in children under 15 years per 100,000 inhabitants	M	2001–2003	28.5	-8.9–81.4	2003–2014	-3.3 ^a^	-5.3– -1.3	-1.4	-3.7–1.0	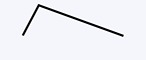
	F	2001–2003	21.6	-15.2–87.6	2003–2014	-4.5 ^a^	-6.8– -2.1	-2.6	-5.1–0.1	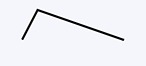
	General	2001–2003	26.1	-6.3–69.6	2003–2014	-3.9 ^a^	-5.8– -1.9	0.2	-3.9–4.5	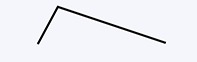
Detection rate with grade 2 disability	M	2001–2014	3.5 ^a^	0.7–6.4				3.5 ^a^	0.7–6.4	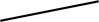
	F	2001–2014	4.6 ^a^	0.7–8.7				4.6 ^a^	0.7–8.7	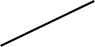
	General	2001–2014	4.0 ^a^	1.3–6.8				4.0 ^a^	1.3–6.8	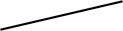
Proportion of multibacillary cases	M	2001–2003	-9.5	-19.7–2.1	2003–2014	3.6 ^a^	2.8–4.5	1.5	-0.2–3.3	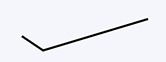
	F	2001–2004	-4.2	-9.9–1.9	2004–2014	5.0 ^a^	4.0–6.0	2.8 ^a^	1.4–4.2	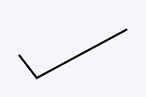
	General	2001–2003	-7.9	-20.3–6.4	2003–2014	4.1 ^a^	3.1–5.1	2.2 ^a^	0.1–4.3	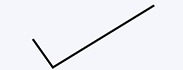

APC: annual percentage change; AAPC: average annual percentage change

^a^ Significantly different from 0 (p < 0.05).

^b^ New case detection rates of new cases, general population (per 100,000 inhabitants).

The new case detection rate among children < 15 years in 2014 was 5.2 cases/100,000 inhabitants, with a mean standard value of 6.1 cases/100,000 inhabitants, being the detection rate higher in 2004 (8.0 cases/100,000 inhabitants) (Figure 3, B). The Joinpoint regression analysis showed a trend of maintenance of the detection among children < 15 years for the state of Bahia, with no statistical significance (AAPC = 0.2, 95%CI -3.9–4.5) (Table).

The average proportion of cases with G2D evaluated at the diagnosis of leprosy was 86.2%. We verified an increase in the proportion of NC with G2D at diagnosis, which increased from 4.3% in 2001 to 7.7% in 2014. We observed 0.6 cases with G2D per 100,000 inhabitants in 2001, increasing to 1.1 cases with G2D per 100,000 inhabitants in 2014 (Figure 3, C).

Since 2008, a higher proportion of multibacillary cases has been identified, accounting for 51.6% of the average of the fourteen years of the period, going from 48.1% in 2001 to 62.3% in 2014 (Figure 3, D). The regression analysis confirmed a significant increasing trend after 2003 (APC = 4.1, 95%CI 3.1–5.1) (Table).

On the other hand, between 2007 and 2012 the new case detection rates among men and women, showed almost identical values in 2013 and 2014, of 17.6/100,000 and 17.1/100,000 inhabitants, respectively (Figure 3, A). The time trend until 2004 showed a significant increase in both genders, being it more marked among women (APC = 15.9, 95%CI, 3.8–29.4) than among men (APC = 16.5, 95%CI 2.8–32.0). Similarly, for the reduction that marks the following years, the slope of the line is higher for women, including new cases in children under 15 years (Table).

The proportion of NC of leprosy with G2D at diagnosis was 78.1% (n = 2,603) among men. The analysis of the detection rate of NC with G2D in relation to the general population shows that, for both genders, there is a trend of increase, but with significant difference among women. Figure 3, C shows that the highest detection rate of NC with G2D occurred in the male population in all periods analyzed. However, throughout the state, the trend of increase was more significant among women (AAPC = 2.8, 95%CI 1.4–4.2) compared to men (AAPC = 1.5, 95%CI -0.2–3.3) (Table).

Most of the multibacillary cases (60.6%) occurred among men, reaching the highest proportion in 2014 (62.3%) (Figure 3, D). After 2003, there was a significant increase in both genders but more pronounced among women (APC = 5.0, 95%CI, 4.0–6.0). Considering the entire period, we identified a trend of increase among women and an maintenance of the trend of this indicator among men (Table).

## DISCUSSION

This study systematically presents the epidemiological context of leprosy in in the state of Bahia during an extensive historical series of fourteen years. The endemic disease persists at high levels and with unequal patterns of expression over time. These trends, associated with the current persistent transmission and morbidity, late diagnosis, and probable hidden endemic (indicated by the high percentage of multibacillary cases and cases with visible disabilities), emphasize the relevance of leprosy as an ongoing public health problem in the state. The gender distribution is differentiated despite the similarity regarding the magnitude of the endemic disease, calling attention to early diagnosis and treatment with specific approaches for men and women. The increasing trends of indicators was more pronounced in the female gender.

The state of Bahia, according to the Brazilian Ministry of Health persists at high levels of high endemicity (between 20.0 to 39.9 new cases annually/100,000 inhabitants)^c^. The patterns of the endemic disease in the different regions confirm a heterogeneous distribution, usually associated with social vulnerability. In fact, unequal risks for the occurrence of leprosy may be associated with demographic, genetic, environmental, socioeconomic, and cultural factors superimposed geographically overlapping the observed patterns of occurrence^3^ ^,^ ^11^ ^,^ ^18^ ^,^ ^19^. This study reaffirms that poor living conditions and the lack of access to health services are associated with the distribution of leprosy in an endemic area^14^ ^,^ ^22^.

The fact that the disease is more frequent among those with lower educational level^5^ ^,^ ^9^, considering this variable as an indicator of living conditions, also reinforces the characteristic as a Neglected Tropical Disease. A study carried out in the state of Minas Gerais, Brazil, has suggested an association between leprosy and low HDI values^14^, which increases the challenges for control. Consistent with this, the states in the Northeast region, marked by high social inequality, show a more pronounced impact of endemic diseases on the health status of the most vulnerable populations^19^.

Although the trend of decrease was significant for the new case detection rate in the state after 2004, the indicator was stable considering the entire period. The year 2004 marked an important period in the Brazilian Unified Health System (SUS) of Bahia, with expansion of the population coverage of the primary health care services, going from 16.8% to 32.3% of the population ^g^. A study about the impact of the Family Health Strategy (FHS) on the control of leprosy, carried out in 1,358 Brazilian municipalities, has indicated a decrease of the detection rate in municipalities with higher coverage of income transfer programs and primary health care coverage^22^. However, the decrease of registered new cases was not sufficient for Bahia to be re-classified from high endemicity, even when reaching 71.7% of population coverage by the FHS in 2014^g^.

New case detection rate in children <15 years still persisted at high endemicity (5.0 to 10.0/100,000 inhabitants)^c^. This unfavorable condition is present in another twelve Brazilian states, that representing 44.0% of the cases nationwide in 2014^a^. Similarly to Bahia, a study carried out in Mato Grosso, Tocantins, Rondônia, Pará, and Maranhão (most endemic states in Brazil), has identified a significant increase in the new case detection rates in children after 2003^3^ ^,^ ^10^. In approximately thirty municipalities of Bahia, campaigns were carried out in 2003 and 2004 and repeated in 2008 and 2009 (Personal Communication, Maria Leide Wand-Del-Rey de Oliveira), which may have contributed to the trends observed. Recently, among the main strategies defined by the Ministry of Health to face leprosy and soil-transmitted helminth infections (2012–2015) that may have contributed to the trends observed in this study, we can mention the development of the “National Leprosy and Geohelminthiasis Campaign” in March of 2013 throughout Brazil. The campaign initially targeted 9,300,000 students in the age group of 5 to 14 years, in approximately 38,000 schools in the 720 priority municipalities.

The occurrence of leprosy in children, even after the introduction of multidrug therapy in 1991^4^, reaffirms the maintenance of active transmission foci with early exposure to *Mycobacterium leprae* ^9^. Possible explanations for this situation include the difficulty of clinical diagnosis, which is accentuated in this period of life, stigma related to the disease, and the fragility of the health promotion and education process in the territories^13^ ^,^ ^17^ ^,^ ^24^. The expansion of primary health care coverage and the effective decentralization of control measures in the health care network were not sufficient to reverse the increasing trend of growth over the period evaluated. A study carried out in Fortaleza, a municipality with significant detection of cases in children, has concluded that the primary health care units did not present a satisfactory performance for diagnosis in this group^2^. A similar study in the Jequitinhonha Valley region, state of Minas Gerais, has shown limited performance of health services, which were passively receiving the demand that comes to services, without systematic active case detection strategies^13^, leading to the diagnosis of more advanced and severe forms of the disease.

The grade of physical disability at diagnosis places Bahia within the parameters considered of medium severity^c^, with almost 8% in 2014. This percentage is higher than the Brazilian (6.5%) and the Northeast averages (5.6%) for the same year. Studies carried out in endemic states, which have registered 34.5% of the Brazilian cases, has shown that the new case rate with G2D remained stable^10^. The state of Bahia presented a significant increase for this rate, which is different from the trend of stability identified in the municipality of Fortaleza from 2001 to 2012^7^ and decrease in the state of Paraíba from 2012 to 2014^8^. If we had verified a trend of decrease of the general detection rate of NC along with the decrease of this rate of NC with G2D, it would characterize the reduction of the endemic in the state.

Because it is an indicator that reflects the effectiveness of timely or early activities of detection of cases^c^, its increase indicates the need to improve and expand the access of the population, especially those with higher social vulnerability^16^, for prevention measures, diagnosis, early treatment, and rehabilitation. As a chronic condition, with the possibility of developing disabilities even after discharge, leprosy care needs longitudinal and integrated measures. The reactional episodes that occur in 20% to 40% of the diagnosed cases before, during, and after the multidrug therapy reinforce these issues^6^ ^,^ ^18^. However, the fragility after release from multidrug therapy in the comprehensive care of persons affected by leprosy^6^ increases the vulnerability to new lesions and disabilities. Other studies also indicate a significant association between the development of G2D and a lower educational level among the persons affected by leprosy, with higher risk for neural impairment and difficulty in moving to the health unit, besides maintaining G2D^25^.

The G2D at diagnosis also represents an indirect marker of a hidden endemic situation, which allows the estimation of the prevalence that is the closest to the real scenario of the disease in a given area. This analysis becomes important in order to define local strategies based on evidence an essential condition for the state of Bahia. Another important issue is the need to follow the routines established by the national guidelines for the achievement and adequate recording of the Eye, Hand, and Foot (EHF) score, at least minimally at diagnosis, throughout treatment, and during discharge^c^. The high proportion of cases with no information on G2D at discharge hindered a more consistent analysis of this important indicator for the entire state of Bahia in this study.

The higher occurrence of multibacillary cases (62%) in relation to the total number of cases, with a significant increase in the state of Bahia, reinforces the epidemiological magnitude of this endemic disease. Brazil reached a percentage of 65.9% in 2014, while the Northeast region, reached 62.3%^a^. When analyzing the therapeutic itinerary of persons with leprosy in the municipality of Salvador, state of Bahia, in 2014, researchers have reported that both the lack of training of health professionals to diagnose early the disease early and stigma and prejudice, which favor the silence around the disease and self-medication, represent some of the reasons identified. This study has also shown that the average time for diagnosis was inadequate, and there were situations in which confirmation of the disease occurred only when the suspected case was referred to the referral center in the state capital^17^.

Although the gender distribution did not show any significant difference, leprosy expression between the genres was not significant, leprosy cases prevailed in Brazil among the male population^a^, similarly to some other studies^5^, including in Bahia, and in 81% of the federative units of Brazil in 2014^a^. This higher occurrence is probably linked to the increased exposure from work-related activities, poor health care demand, poor self-care, and reduced access to information. Therefore, gender must be recognized as an important determinant for the occurrence and severity of the disease, especially when the general pattern of increased health risk among men is considered. The way men perceive and use their bodies generates specific needs, including health access and protection^23^ ^,^ ^26^.

The analysis of the new case detection trend indicates, as of 2004, the maintenance of this rate among men and reduction among women. When compared to men, women had a more expressive increase of the proportion of multibacillary cases. On the other hand, in absolute values, almost 80% of the cases diagnosed with G2D were males. However, the trend of increase of the rates of cases with G2D was higher among women. We can infer that the higher increase of NC of leprosy among women refers to the lower barrier for diagnosis when compared to men. Among the male population, late diagnosis or lack of diagnosis seems to be more frequent, contributing to the hidden endemic scenario. Although the outcome of the trend analysis is favorable for women, the occurrence of multibacillary and G2D cases should be considered as a serious situation, as it may indicate hidden prevalence.

Schraiber et al.^26^ have affirmed that “the inclusion of the participation of men in actions of health is at least a challenge for different reasons. One of them refers to the fact that, in general, taking caring of oneself and appreciating the body in the health sense, also regarding taking care of others, are not issues placed in the socialization of men” (p.8). Existing barriers need to be recognized and overcome for better access and effectiveness of the leprosy control measures from a gender perspective. One of the paths should be the analysis of the singularities of men and women in society^23^. In addition to the care and attention to men’s health, prevention measures, early diagnosis, and prompt treatment for this population should be understood as essential for caring of persons, families, and communities^26^ ^,^ ^27^ and for the reduction of the transmission of *M. leprae* in Bahia. Therefore, the actions of the National Men’s Health Policy need to be structured more consistently and broadly.

This study presents limitations regarding the use of secondary databases, considering the non-completeness and inconsistencies of some variables. However, the incorporation of the state database in a historical series of fourteen years, together with the need for studies with this approach in the state of Bahia, justifies its use.

## CONCLUSIONS

Leprosy persists in Bahia, throughout the historical series analyzed, as a significant public health problem, with a high magnitude and a still limited decrease of the new case detection rate. These transmission dynamics are evidenced by the maintenance of high new case detection rates in children. On the other hand, transcendence remains in view of the high and sustained proportion of NC of leprosy with G2D at diagnosis. The endemic behavior highlights the higher vulnerability among men from the gender perspective, which contributes to the maintenance of the dynamics of transmission of *M. leprae*. However, it is necessary to monitor and study the reasons for late diagnosis in women.

There are great challenges for the control of diseases linked to poverty, social inequalities, and health inequities. Profound reforms that address the complex social determinants of leprosy are required to face these challenges^9^ ^,^ ^11^. Economic, cultural, and social changes are necessary to reduce the different dimensions of vulnerability in family groups affected by the disease.

In addition, the improvement of control measures developed by the services of the SUS network should always be sought^6^ ^,^ ^22^ ^,^ ^24^. This study indicates the need for new research that addresses the dynamics of transmission in historically high endemic areas and the reasons for the low resolution of health services for timely prevention, diagnosis, and treatment, also from a gender perspective. The development of operational health research studies is strategic for the strengthening of care networks^24^, especially when we consider the complexity of elimination as a goal for leprosy control as a public health problem^24^ ^,^ ^28^ ^,^ ^29^.

Finally, we reiterate the need to develop actions systematically aimed at health education, paying attention to specific gender issues..

## Footnotes

a Ministério da Saúde (BR), Secretaria de Vigilância em Saúde, Departamento de Vigilância das Doenças Transmissíveis. Apresentação durante o 8 Simpósio Brasileiro de Hansenologia, São Paulo, 2015.

b Ministério da Saúde (BR), Secretaria de Vigilância em Saúde, Departamento de Vigilância das Doenças Transmissíveis. Plano integrado de ações estratégicas de eliminação da hanseníase, filariose, esquistossomose e oncocercose como problema de saúde pública, tracoma como causa de cegueira e controle das geohelmintíases. Plano de Ação 2011-2015. Brasília (DF); 2012 [cited 2016 Sep 12]. (Série C. Projetos, Programas e Relatórios). Available from: http://bvsms.saude.gov.br/bvs/publicacoes/plano_integrado_acoes_estrategicas_2011_2015.pdf

c Ministério da Saúde (BR), Secretaria de Vigilância em Saúde, Departamento de Vigilância das Doenças Transmissíveis. Diretrizes para vigilância, atenção e eliminação da hanseníase como problema de saúde pública: manual técnico-operacional. Brasília (DF); 2016 [cited 2016 Sep 12]. Available from: http://portalsaude.saude.gov.br/images/pdf/2016/fevereiro/04/diretrizes-eliminacao-hanseniase-4fev16-web.pdf

d Instituto Brasileiro de Geografia e Estatística. Unidades da Federação: Bahia. Rio de Janeiro: IBGE; 2014 [cited 2016 Sep 10]. Available from: https://ww2.ibge.gov.br/estadosat/perfil.php?sigla=ba

e Federação das Indústrias do Estado do Rio de Janeiro. Índice da Federação das Indústrias do Estado do Rio de Janeiro de Desenvolvimento Municipal. Rio de Janeiro; 2014 [cited 2016 Sep 10]. Available from: http://www.firjan.com.br/ifdm/

f Programa das Nações Unidas para o Desenvolvimento; Instituto de Pesquisa Econômica Aplicada; Fundação João Pinheiro. Atlas do desenvolvimento humano no Brasil 2013. Brasília (DF); 2013 [cited 2016 Sep 10]. Available from: http://atlasbrasil.org.br/2013/pt/perfil_uf/bahia

g Ministério da Saúde (BR), Secretaria de Atenção à Saúde, Departamento de Atenção Básica. Histórico de cobertura da Saúde da Família. Brasília (DF); 2016 [cited 2016 Sep 2]. Available from: http://dab.saude.gov.br/portaldab/historico_cobertura_sf.php

## References

[1] Adhikari B, Kaehler N, Chapman RS, Raut S, Roche P (2014). Factors affecting perceived stigma in leprosy affected persons in western Nepal. PLoS Negl Trop Dis.

[2] Alencar CH, Barbosa JC, Ramos AN, Alencar MJF, Pontes RJS, Castro CGJ (2008). Hanseníase no município de Fortaleza, CE, Brasil: aspectos epidemiológicos e operacionais em menores de 15 anos (1995-2006). Rev Bras Enferm.

[3] Alencar CH, Ramos AN, Barbosa JC, Kerr LR, Oliveira ML, Heukelbach J (2012). Persisting leprosy transmission despite increased control measures in an endemic cluster in Brazil: the unfinished agenda. Lepr Rev.

[4] Andrade V (2006). Implementação da PQT/OMS no Brasil. Hansenol Int.

[5] Barbosa DRM, Almeida MG, Santos AG (2014). Características epidemiológicas e espaciais da hanseníase no Estado do Maranhão, Brasil, 2001-2012. Medicina.

[6] Barbosa JC, Ramos AN, Alencar OM, Pinto MSP, Castro CGJ (2014). Atenção pós-alta em hanseníase no Sistema Único de Saúde: aspectos relativos ao acesso na região Nordeste. Cad Saude Coletiva.

[7] Brito AL, Monteiro LD, Ramos AN, Heukelbach J, Alencar CH (2016). Temporal trends of leprosy in a Brazilian state capital in Northeast Brazil: epidemiology and analysis by joinpoints, 2001 to 2012. Rev Bras Epidemiol.

[8] Brito KKG, Andrade SSC, Santana EMF, Peixoto VB, Nogueira JA, Soares MJGO (2015). Epidemiological analysis of leprosy in an endemic state of northeastern Brazil. Rev Gaucha Enferm.

[9] Cabral-Miranda W, Chiaravalloti F, Barrozo LV (2014). Socio-economic and environmental effects influencing the development of leprosy in Bahia, north-eastern Brazil. Trop Med Int Health.

[10] Freitas LR, Duarte EC, Garcia LP (2016). Trends of main indicators of leprosy in Brazilian municipalities with high risk of leprosy transmission, 2001-2012. BMC Infect Dis.

[11] Imbiriba ENB, Silva AL, Souza WV, Pedrosa V, Cunha MG, Garnelo L (2009). Social inequality, urban growth and leprosy in Manaus: a spatial approach. Rev Saude Publica.

[12] Kim HJ, Fay MP, Feuer EJ, Midthune DN (2000). Permutation tests for joinpoint regression with applications to cancer rates. Stat Med.

[13] Lana FCF, Amaral EP, Lanza FM, Lima PL, Carvalho ACN, Diniz LG (2007). Hanseníase em menores de 15 anos no Vale do Jequitinhonha, Minas Gerais, Brasil. Rev Bras Enferm.

[14] Lana FCF, Davi RFL, Lanza FM, Amaral EP (2009). Detecção da hanseníase e Índice de Desenvolvimento Humano dos municípios de Minas Gerais. Brasil. Rev Eletr Enf.

[15] Laurenti R, Jorge MHPM, Gotlieb SLD (2005). Perfil epidemiológico da morbi-mortalidade masculina. Cienc Saude Coletiva.

[16] Lopes VAS, Rangel EM (2014). Hanseníase e vulnerabilidade social: uma análise do perfil socioeconômico de usuários em tratamento irregular. Saude Debate.

[17] Martins PV, Iriart JAB (2014). Itinerários terapêuticos de pacientes com diagnóstico de hanseníase em Salvador, Bahia. Physis.

[18] Martins-Melo FR, Assunção-Ramos AV, Ramos AN, Alencar CH, Montenegro RM, Wand-Del-Rey de Oliveira ML (2015). Leprosy-related mortality in Brazil: a neglected condition of a neglected disease. Trans R Soc Trop Med Hyg.

[19] Martins-Melo FR, Ramos AN, Alencar CH, Heukelbach J (2016). Mortality from neglected tropical diseases in Brazil, 2000-2011. Bull World Health Organ.

[20] Monteiro LD, Martins-Melo FR, Brito AL, Alencar CH, Heukelbach J (2015). Physical disabilities at diagnosis of leprosy in a hyperendemic area of Brazil: trends and associated factors. Lepr Rev.

[21] Monteiro LD, Martins-Melo FR, Brito AL, Alencar CH, Heukelbach J (2015). Spatial patterns of leprosy in a hyperendemic state in Northern Brazil, 2001-2012. Rev Saude Publica.

[22] Nery JS, Pereira SM, Rasella D, Penna ML, Aquino R, Rodrigues LC (2014). Effect of the detection rate of leprosy. PLoS Negl Trop Dis.

[23] Oliveira MHP, Romanelli G (1998). Os efeitos da hanseníase em homens e mulheres: um estudo de gênero. Cad Saude Publica.

[24] Ramos AN, Heukelbach J, Gomide M, Hinders DC, Schreuder PA (2006). Health systems research training as a tool for more effective Hansen’s disease control programmes in Brazil. Lepr Rev.

[25] Ribeiro GC, Lana FCF, Diamantina M (2015). Incapacidades físicas em hanseníase: caracterização, fatores relacionados e evolução. Cogitare Enferm.

[26] Schraiber LB, Gomes R, Couto MT (2005). Homens e saúde na pauta da Saúde Coletiva. Cienc Saude Coletiva.

[27] Schraiber LB (2012). Healthcare needs, public policies and gender: the perspective of professional practices. Cienc Saude Coletiva.

[28] Smith CS, Aerts A, Kita E, Virmond M (2016). Time to define leprosy elimination as zero leprosy transmission?. Lancet Infect Dis.

[29] Smith WC, Brakel W, Gillis T, Saunderson P, Richardus JH (2015). The missing millions: a threat to the elimination of leprosy. PLoS Negl Trop Dis.

